# Impact of Gender-Affirming Surgery on Psychiatric Outcomes and Quality of Life in Transgender Individuals: A Systematic Review of Longitudinal Cohort Studies

**DOI:** 10.3390/jcm15062213

**Published:** 2026-03-14

**Authors:** Keith A. Yeo, Yi Jie Yeo, Cyrus Su Hui Ho

**Affiliations:** 1 Department of Psychological Medicine, National University Hospital, Singapore 119228, Singapore; yijie.yeo@mohh.com.sg (Y.J.Y.); pcmhsh@nus.edu.sg (C.S.H.H.); 2 Department of Psychological Medicine, Yong Loo Lin School of Medicine, National University of Singapore, Singapore 119077, Singapore

**Keywords:** transgender, gender dysphoria, gender-affirming surgery, psychiatric outcomes, quality of life

## Abstract

**Background/Objectives:** Gender-affirming surgery (GAS) has become more accessible in recent years. It aims to align the physical characteristics of transgender individuals with their gender identity to alleviate distress associated with gender dysphoria. This may involve procedures such as genital reconstruction, breast augmentation or removal, and voice modification surgeries. However, the associations of these treatments on long-term mental health outcomes remain debated. This paper aims to review and synthesize current research on the associations of GAS on psychiatric outcomes and quality of life in transgender individuals. **Methods:** In accordance with the PRISMA statement, a search on PubMed, PsychInfo, and Embase yielded 867 articles, of which 14 studies of 3023 participants met the full inclusion criteria. **Results:** There is an initial improvement in psychological well-being and quality of life within the first year post-GAS, followed by subsequent plateau or decline thereafter. Factors such as younger age, higher levels of education, noticeable improvement in secondary sexual characteristics, and a supportive social environment have been identified as predictors of positive outcomes. Conversely, non-homosexual orientation and higher levels of pre-GAS psychopathology have been associated with poorer outcomes, highlighting the importance of tailored support and pre-operative mental health care to optimize long-term success. **Conclusions:** This study underscores the need for further research into long-term outcomes and tailored support strategies to optimize the mental health and well-being of transgender individuals undergoing GAS.

## 1. Introduction

Gender dysphoria, characterized by the significant distress experienced when an individual’s gender identity is incongruent with their physical phenotype or societal gender role, is a relatively rare condition. Current prevalence rates reported in the literature, estimated at less than 0.1% for both male and female populations, are likely underreported, as many individuals with gender dysphoria do not seek treatment or disclose their experiences [1]. However, there has been a notable global increase in referrals to mental health services for gender dysphoria in recent years, reflecting heightened awareness, greater access to care, and evolving societal attitudes toward gender diversity [1]. Recent studies investigating possible reasons for this rising trend have acknowledged a likely multifactorial basis such as increased societal recognition, acceptance of gender diversity, and the impact of social media, amongst others [2].

Gender dysphoria is also associated with significant comorbidity. Depression, anxiety and suicide rates are higher than those in the general population, and correspondingly, wider consequences of economic marginalization, unemployment, and discrimination are observed [3]. Recognizing the consequences on these individuals, support programs and gender dysphoria clinics have been established over the years, offering assessment and counseling support services, as well as surgical and medical treatment to align their physical characteristics with their gender identity.

The landscape of gender treatment has continued to evolve over the years with increasing accessibility of and advancements in surgical techniques and medical treatment. Gender-affirming surgery (GAS) is a key medical intervention that primarily aims to facilitate affirmation and congruence with one’s gender identity. However, the extent to which GAS is associated with psychological outcomes and quality of life remains a subject of ongoing research with varying results in the studies that have looked into the psychological outcomes of gender treatment. Some have reported significant improvements in measures of psychological outcomes and quality of life, while others have indicated that it may not fully resolve all psychological issues and have highlighted the need for continued mental health support post-treatment. A critical examination and synthesis of the current body of research would help to further understanding of the benefits and limitations of these interventions so as to refine clinical approaches to ensure these individuals receive appropriate and effective care.

## 2. Methodology

To align with current clinical and scientific standards, this manuscript utilizes the terms transgender women and transgender men instead of the historical binary labels (MTF/FTM) and employs gender-affirming surgery (GAS) as the encompassing term. We acknowledge that the original studies included in this review may have used the older terminology; however, we have adopted the current language for interpretative clarity.

### 2.1. Data Search

The literature search and review protocol were designed and performed in accordance with the Preferred Reporting Items for Systematic Reviews and Meta-Analyses (PRISMA) statement, although they were not registered [4]. The PRISMA Checklist is included in the Supplementary Table S1. A systematic search using PubMed, EMBASE, and Psychinfo was conducted on 12 March 2024 using the search terms (“sex reassignment surgery” OR “gender reassignment surgery”) AND (“gender dysphoria” OR “transgender persons”) and MeSH terms relating to outcomes including quality of life, suicide, hospitalization, depression, anxiety, personality disorder, substance use disorder, post-traumatic stress disorder, and psychological symptoms. Endnote 20 was used to manage citations and resolve duplicates. Back-referencing was used to identify potential studies and relevant citations to be included in our analysis. The search yielded 867 articles.

### 2.2. Eligibility Criteria

The inclusion and exclusion criteria were developed a priori. The main inclusion criteria necessitated the inclusion of individuals diagnosed with gender dysphoria who had undergone GAS. Additionally, studies were required to specifically assess the change in quality of life or psychiatric comorbidities both before and after sex reassignment surgery. Lastly, studies were limited to those published in the English language. We avoided looking at outcomes such as sexual function, body gender congruence score, and body satisfaction scores as we were primarily concerned with broader quality of life measurement and other psychological comorbidities as opposed to dimensions of gender dysphoria or urological function. Multiple studies do show a marked increase in such outcomes after GAS [5,6].

### 2.3. Data Selection Process

Two independent reviewers screened titles and abstracts of identified records to determine eligibility. Full-text articles of potentially relevant studies were retrieved and assessed for final inclusion. Any reviewer disagreements were resolved through discussion or consultation with a third reviewer. Two independent reviewers assessed the quality of the included studies via the Newcastle–Ottawa Scale for Cohort Studies [7]. Data extraction tables were used to register the key characteristics of the included studies and the following variables were registered: author, year, participants, mean age of participants (year), type of surgery, length of follow-up, outcome measured, instruments used, significant findings, and possible predictors of good outcomes. The complete, database-specific Boolean search strings, including all syntax and field tags, are detailed in the Supplementary Table S2.

### 2.4. Study Quality

Included studies were rated using the Newcastle–Ottawa Scale for Cohort Studies (NOS). The scale rates studies with up to 4 points for Selection, 2 points for Comparability, and 3 points for Outcomes, with scores equal to or greater than 7 considered high quality and scores less than 7 considered low quality. We took 1 year as an adequate follow-up time and a response rate or loss to follow-up rate of 50% and below as poor.

## 3. Results

### 3.1. Search Results

**Figure 1:**
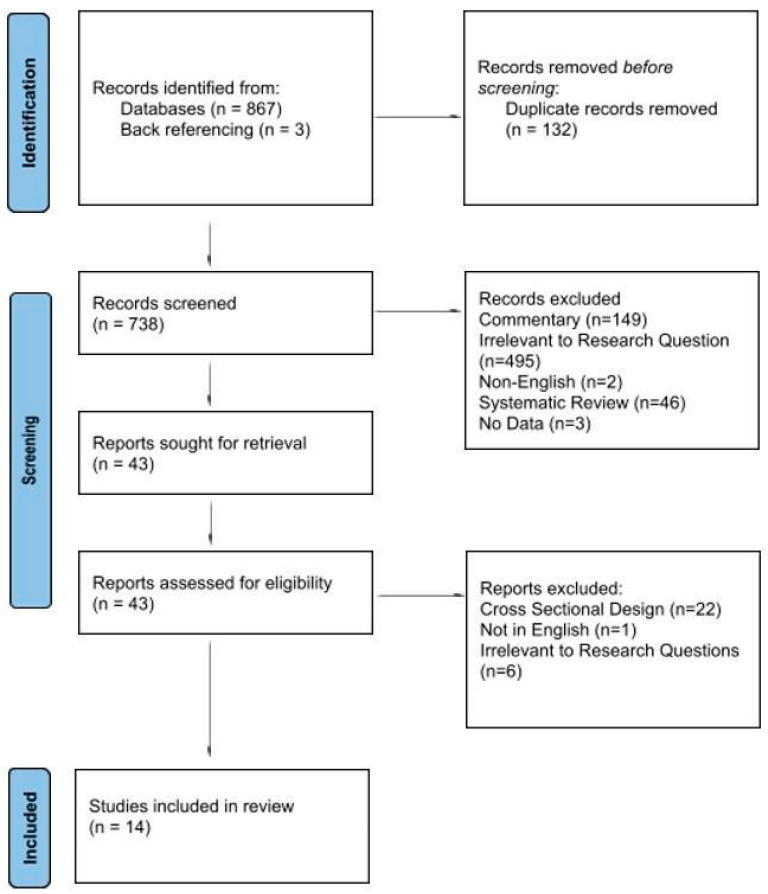
PRISMA flowchart.

The search was conducted on 12 March 2024; the initial search identified 867 studies, of which 132 were duplicates and 693 were excluded before full-text review. The PRISMA flowchart is shown in Figure 1. The remaining 40 had their abstract and full text reviewed, and a large majority were further excluded for having a cross-sectional design rather than a cohort design. Three additional studies were included during back-referencing, leading to a total of 13 studies and one conference abstract. These studies represented a total of 3023 individuals who had undergone GAS, of which 631 had some form of masculinizing GAS and 1374 had some form of feminizing GAS.

### 3.2. Characteristics of Participants

The mean age of the participants at the time of receiving GAS from each study ranged from 16 to 47.33 years old. At least nine studies involved genital surgery, four studies did not explicitly specify the nature of the surgery, while one study [8] focused exclusively on chest reconstruction. The longest follow-up was for up to 13 years [9], while the shortest follow-up was 6 months post-GAS [10].

### 3.3. Outcomes and Tools

The most common outcome measured was quality of life, with both the Short-Form Health Survey (SF-36) and variants of the World Health Organization Quality of Life Scale (WHOQOL) scales being used, and one study [11] used the Affect Balance Scale. Gender dysphoria was the next most common outcome measured, conducted using the Utrecht Gender Dysphoria Scale (UGDS). Only three studies measured psychiatric outcomes such as changes in psychiatric diagnosis, number of hospitalizations after suicide attempts and inpatient psychiatric encounters. Three studies also measured psychological symptoms using the Symptom Checklist-90 (SCL-90) tool or variants of it.

### 3.4. Quality of Life

A summary of the key findings of the included studies are presented in Table 1.

Most studies reported a general improvement in QOL post-GAS. Lindqvist et al. [12] noted a non-significant decline in QOL at 5 years post-GAS. da Silva et al. [13] noted a significant decline in physical health and level of independence after GAS, whereas Naeimi et al. [14] showed a significant improvement in all dimensions of the SF-36, including physical health 6 months post-GAS. Smith et al. [11] and de Vries et al. [15] did not compare QOL before and after GAS. de Vries et al. [15] compared the QOL scores from large validation studies, which revealed largely similar scores. Smith et al. [11] could not find a comparison group of similar age. Becker-Hebly et al. [16] showed improvements in health-related QOL post-GAS, though no significance testing was done.

**Table 1:** Table of study characteristics.

No.	Author, Year	Participants	Age (Year)	Type of Surgery	Length of Follow Up	Outcome Measured	Instruments Used	Significant Findings
1	Agarwal et al., 2018 [8]	42 transgender men	27.7	Chest reconstruction	6 months after GAS	Breast satisfaction, psychosocial well-being, sexual satisfaction, physical well-being, body image	BREAST-Q BUT-A	Post-GAS surveys showed statistically significant improvements in breast satisfaction, psychosocial well-being, sexual satisfaction, and physical well-being. BUT-A results showed significant decreases in avoidance behaviors, compulsive self-monitoring, and depersonalization post-GAS. The improvement in body image was particularly significant for those with pre-existing mental health conditions.
2	Bränström et al., 2020 [17]	1018; did not specify transgender women or transgender men	31.5	Chest, genital, dermatological and laryngeal surgery	1–10 years after GAS	Psychiatric outpatient care, healthcare visits, antidepressant and anxiolytic prescriptions, and hospitalization after a suicide attempt.	Direct measurements of records	The risk of mental health treatment post-GAS significantly decreased with an increase in time since the last surgical treatment. Adjusted OR was 0.92 (95% CI = 0.87, 0.97). The likelihood of being treated for a mood or anxiety disorder was reduced by 8% for each year since the last GAS. The association between time since hormone and surgical treatments and hospitalization after a suicide attempt did not reach statistical significance.
3	da Silva et al., 2016 [13]	47 transgender women	31.2	Penile inversion vaginoplasty	At least 12 months after GAS	Quality of life	WHOQOL-100	Domains II (psychological) and IV (social relationships) of WHOQOL-100 showed significant improvement after GAS. However, domains I (physical health) and III (level of independence) were significantly worse after GAS.
4	Dallas et al., 2021 conference abstract [18]	869 transgender women and 357 transgender men	Not stated	Genital surgery	Mean of 2 years after GAS	Psychiatric emergencies	Emergency room and psychiatric inpatient encounters	Overall risk for psychiatric encounters and suicide were increased after GAS for transgender women but decreased for transgender men, though there was no mention of statistical significance. The rate of a psychiatric encounter occurring post-GAS if an episode pre-GAS occurred was 33.9% and 26.5% for the transgender women and transgender men groups respectively. The overall rates of suicide attempts doubled (3.3% vs. 1.5%, *p* = 0.017) after feminizing GAS; such an effect was not observed after masculinizing GAS.
5	Simonsen et al., 2015 [9]	56 MTF and 48 transgender men	27.7	Genital surgery	Mean of 13 years after GAS	Psychiatric diagnosis	Direct measurements of records	No notable distinctions were observed in the prevalence of psychiatric morbidity between transgender women and transgender men pre- and post-GAS. However, the transgender men group exhibited a significantly higher overall presence of psychiatric diagnoses. About 7% of the sample received psychiatric diagnoses both before and after GAS, indicating consistent psychiatric morbidity throughout this period.
6	De Vries et al., 2014 [15]	22 transgender women and 33 transgender men	20.7	Genital surgery (excluding phalloplasty for transgender men)	Mean of 12 months after GAS	Gender dysphoria/body image, psychological functioning, quality of life, and objective and subjective well-being	UGDS, CGAS, WHOQOL-BREF, SHS, SWLS Non-standardized questionnaire for specific inquiries WHOQOL-BREF was only done after GAS	Subjective well-being measures (WHOQOL-BREF, SWLS, SHS) indicated overall well-being comparable to same-age peers. Psychological functioning showed steady improvement over time. CGAS, BDI and behavioral/emotional problem scores indicated positive changes. Some quadratic trends suggested initial decreases followed by increases in depression and internalizing problems from after hormonal treatment to post-GAS. WHOQOL-BREF’s Environment subdomain was better than the standardization sample. Authors attributed this to supportive social and financial factors in the Netherlands.
7	Lindqvist et al., 2017 [12]	190 transgender women	36	Did not specify	1, 3, and 5 years post-GAS	Quality of life	SF-36	While there was an initial trend towards improved QOL post-GAS, this improvement decreased over time, with a decline in QOL compared to pre-GAS at the 5 year mark, although not statistically significant. Results possibly confounded by loss to follow up and with only those having poorer outcomes remaining to complete the survey. Lower QOL in transgender women compared to women from the general population.
8	Cohen-Kettenis, 1997 [19]	7 transgender women and 15 transgender men	17.5	Breast removal and genital surgery	Mean of 2.6 years after GAS	IQ, gender dysphoria, body dissatisfaction, and psychological functioning	IQ Tests (WISC-R, WAIS and GIT-2), UGS, BIS, AAI, NPV, NVM, semi-structured oral interviews, social reaction questionnaire	Significant decrease in gender dysphoria post-GAS. Significant increase in body satisfaction with primary and sexual characteristics post-GAS. Significant increase in extroversion on NVM post-GAS. Significant increase in dominance and self-esteem and a significant decrease in inadequacy on NPV post-GAS.
9	Smith, Y. L. S. et al., 2001 [11]	7 transgender women and 13 transgender men	16.6	Breast removal and genital surgery	1–4 years before and 1–7 years after GAS	Gender dysphoria, body dissatisfaction, psychological functioning, quality of life	UGDS, BIS, AAI, DSM, SCL-90, semi-structured interviews, self-developed questionnaires, and Affect Balance Scale. Only the UGDS, BIS, AAI and Personality Questionnaires were done both pre- and post-GAS.	Reported less gender dysphoria (*p* < 0.001) post-GAS. NVM of the treatment group showed no significant changes post-GAS. Both pre- and post-test scores were within the average range of Dutch normative data. The mean treatment group’s total score on the SCL-90 (psychoneuroticism) was not significantly lower post-GAS than pre-GAS. There was a decrease in anxiety, depression, and hostility scores from the pre-GAS to the post-GAS assessment on SCL-90.
10	B. Udeze et al., 2008 [10]	40 transgender women	47.33	Did not specify	6 months after GAS	Psychological symptoms	SCL-90 R	GAS had no significant effect on psychological functioning. No difference between SCL scores pre- and post-GAS when compared to a group on waitlist for surgery. Unexplained increases in anger/hostility subscale ratings on SCL-90 R were noted.
11	S. Naeimi et al., 2019 [14]	42 transgender men	34.17	Did not specify	6 months after GAS	Quality of life	SF-36	The study showed a significant increase in QOL scores post-GAS across various domains on the SF-36, except for emotional problems. Significant improvements in physical and psychological health post-GAS, with age and education level significantly influencing these changes.
12	Smith, Y. L. S. et al., 2005 [20]	94 transgender women and 71 transgender men	30.9	Breast augmentation, metoidioplasty, phalloplasty	Mean of 21.3 months	Gender dysphoria, body dissatisfaction, psychological functioning	Social Support Scale, UGS, BIS, AAI, NVM, SCL-90	Gender dysphoria significantly decreased after sex reassignment; post-test scores indicated a near absence of gender dysphoria. Psychological functioning improved post-GAS, with reduced scores on measures of negativism, somatization, shyness, psychopathology, and psychoneuroticism. Comparisons with Dutch normative data showed improvement in psychological stability post-GAS for both transgender women and transgender men.
13	Chaovanalikit et al., 2022 [21]	41 transgender women	26.2	Surgery that reassigns the external genitalia from male to female, including vaginoplasty	6 months after GAS	Quality of life, self-esteem, depression	WHOQOL-BREF-THAI, Rosenberg Self-Esteem Scale, Patient Health Questionnaire-9	Significant improvements in the psychological QOL and self-esteem were reported post-GAS. Five participants met the criteria for mild depression in the pre-GAS, but improved to the point of recovery post-GAS.
14	Inga Becker-Hebly et al., 2020 [16]	1 transgender women and 10 transgender men	16	Mainly breast removal; others not specified	Average of 2 years after GAS	Emotional, behavioral, and social functioning and health-related quality of life	Youth Self Report, Adult Self Report, The Children’s Global Assessment Scale, Kidscreen-27, SF-8	Improvement in health-related QOL and psychological functioning post-GAS was seen, though no testing for statistical significance was done. Only physical QOL was comparable to the German norm at follow-up. Internalizing and externalizing problem scores decreased post-GAS and were comparable to the norm. Clinician-rated CGAS global functioning scores improved post-GAS, though no testing for statistical significance was done.

AAI: Appraisal of Appearance Inventory; BIS: Body Image Scale; BREAST-Q: Breast Surgery Outcome Questionnaire; BUT-A: Body Uneasiness Test-A; CGAS: Children’s Global Assessment Scale; GAS: gender affirming surgery; GIT-2: Groninger Intelligence Test-2; Kidscreen-27: Kidscreen-27 Quality of Life Questionnaire; MMPI: Minnesota Multiphasic Personality Inventory; NVM: Dutch Short MMPI; NPV: Dutch Personality Questionnaire; OR: Odds Ratio; QOL: quality of life; Rosenberg Self-Esteem Scale: Rosenberg Self-Esteem Scale; SCL-90: Symptom Checklist-90; SCL-90 R: Symptom Checklist-90 Revised; SF-36: 36-Item Short Form Survey; SF-8: Short Form-8 Health Survey; SHS: Subjective Happiness Scale; SWLS: Satisfaction with Life Scale; UGDS: Utrecht Gender Dysphoria Scale; WAIS: Wechsler Adult Intelligence Scale; WHOQOL-100: World Health Organization Quality of Life assessment (100 items); WHOQOL-BREF: World Health Organization Quality of Life Assessment (short version); WISC-R: Wechsler Intelligence Scale for Children—Revised.

### 3.5. Dysphoria and Body Image

Most studies reported a significant decrease in gender dysphoria post-GAS. de Vries et al. [15] reported that transgender women had higher satisfaction over time with primary sex characteristics than transgender men, though this is easily explained as none of the transgender men in the paper underwent phalloplasty. Other studies did not report a significant difference between transgender women or transgender men in gender dysphoria post-GAS. This finding aligns with the central objective of gender-affirming procedures: to improve body congruence and facilitate embodiment, the experience of feeling aligned with one’s physical self.

### 3.6. Psychiatric Diagnosis/Measurements and Psychopathology (SCL-90)

Dallas et al. [18] found that the overall risk for suicide attempts was increased after GAS for transgender women but decreased for transgender men. In particular, rates of suicide attempts doubled after vaginoplasty but not after phalloplasty. However, Simonsen et al. [9] noted no changes in the psychiatric diagnosis pre- and post-GAS. Differing from both studies, Bränström and Pachankis [17] observed a significant decrease in mental health treatment utilization with each year following gender-affirming surgery (GAS). However, it is important to note that the association between the time elapsed since hormone therapy and surgical treatments and the risk of hospitalization following a suicide attempt did not reach statistical significance. Additionally, methodological limitations of the study should be considered, as highlighted in the correction to Bränström and Pachankis [17]. Specifically, the study focused on mental health treatment utilization during a single year rather than comparing data across periods before and after GAS, which limits the ability to draw definitive conclusions about long-term outcomes.

Chaovanalikit et al. [21] reported a resolution of all five cases of mild depression that were detected pre-GAS. Smith et al. [11] reported a decrease in anxiety, depression, and hostility scores following GAS using the SCL-90, whereas Udeze et al. [10] reported no significant differences post-GAS using SCL-90 R.

Possible predictors of response to GAS are shown in Table 2.

### 3.7. Study Quality

Study quality is presented in Table 3. Most studies had scores of 5–6 according to the NOS, suggesting poor quality. Only one had a score of 7, indicating high quality. Notably, most studies did not compare samples who underwent GAS to transgender individuals who did not undergo GAS. Some studies used general populations as a comparator, while others did not compare the outcomes to a control population. As such, most studies did not score highly in the Comparability component. There were no notable differences in outcomes correlating with the quality assessment.

## 4. Discussion

### 4.1. Trend of QOL Post-GAS

The findings across the 14 included cohort studies generally indicate that gender-affirming surgery (GAS) is associated with a significant reduction in gender dysphoria and an initial improvement in quality of life (QoL). However, several longer-term studies suggest a pattern of initial QoL gains (peaking 6–12 months post-GAS) followed by plateau or modest decline, although not all differences reached statistical significance.

Most included studies showed positive outcomes within 1 year or 6 months, but looking at more than 1 year post-GAS reveals a more interesting trend. Lindqvist et al. [12] and de Vries et al. [15] showed an initial increase in QOL followed by a gradual decline. Worryingly, Lindqvist et al. [12] showed that QoL at 5 years was lower than pre-GAS levels. In contrast, Bränström and Pachankis [17] showed a trend of a decreasing risk of mental health treatment year on year, which persisted even after more than 10 years. However, on closer inspection, the prevalence of treatment for mood or anxiety disorders decreased by nearly 10% in 1 year, but then increased by 1.2% in 2–3 years post-GAS, followed by a more gradual decline until over 10 years. In addition, the methodology of Bränström and Pachankis [17] has raised concerns, as it focused on mental health treatment utilization during one specific year (i.e., 2015) rather than the periods before and after GAS. A correction was later issued regarding this issue and when compared to a matched control group who did not undergo GAS, no significant improvement was found [22].

Breidenstein et al. [23], in another study not included here, evaluated the long-term results up to 21 years after the GAS and found that although the level of psychosocial resources increased in the first years post-GAS, there was an insignificant increase in the following years. This reinforces what De Cuypere, Elaut [24] suggested: that the first year post-GAS is a ‘honeymoon period’, which does not represent the long-term and emotional status of the patient. Lindqvist et al. [12] has suggested this may be due to disappointment in the long-term outcome of GAS or a reflection that the improvement from GAS plateaued below the QOL of the general population. We posit that the initial psychological benefit derived from surgical congruence (i.e., re-embodiment) is a short-term gain that is gradually undermined by chronic psychosocial stressors over the long term. This underscores that QoL maintenance is possibly dependent on a supportive external environment, not just the physical change brought by GAS itself.

### 4.2. Limited Effects of Improvement in Surgical Techniques

Of the five studies which included GAS done prior to 2010, three studies reported an improvement in QOL or psychological functioning. This was a larger effect than hypothesized; we had expected that poorer surgical techniques would cause fewer improvements post-GAS in older studies, with an improvement seen after the 2010s when advancements in surgical techniques were greater [25]. This likely indicates that surgical technique is less of a factor than other more dynamic issues, such as social acceptance or decreases in gender dysphoria.

### 4.3. Gender-Specific Considerations

While the primary objective of this review was to synthesize the impact of GAS on all transgender individuals, a secondary observation emerged regarding potential disparities in trajectories between transgender men and transgender women. The included papers broadly agree with other studies [26,27] that transgender men may experience more favorable psychiatric and QOL trajectories after GAS than transgender women, although such findings are not uniform across all studies. In an observational study without matched controls, it was noted that there was an association with better outcomes post-GAS; specifically, the risk of suicide attempts for transgender women was increased post-vaginoplasty but was not for transgender men post-phalloplasty [18]. Potential explanations for these findings include residual confounding, such as a higher baseline psychiatric burden in those selected for surgery, surveillance bias, or unmeasured psychosocial stressors.

Studies which exclusively focused on transgender women [10] reported less improvement in post-GAS scores for psychological functioning compared to those with both transgender women and transgender men [11,20]. Two studies showed that QOL for physical health in the short term (6 months to 1 year) was decreased in transgender women [13], though the significance level was not described in one [12]; this finding was not replicated in studies which exclusively involved transgender men [14] or included both transgender women and transgender men [11,15]. An exception to this was the study by Chaovanalikit et al. [21], which reported significant improvements in transgender women across all domains; this positive outcome is posited to be influenced by Thailand’s social norms, which are widely recognized as having a higher visibility of trans people [28].

The poorer outcomes observed in transgender women cannot be solely attributed to surgical factors, as most studies report higher rates of post-surgical complications in transgender men compared to transgender women [29,30,31]. However, these disparities may be better explained by the consistently lower baseline quality of life (QoL) observed in transgender women compared to transgender men [26,32,33]. Supporting this, Chaovanalikit et al. [21] found that pre-GAS QoL data in their sample of transgender women was uniquely above average, which contributed to better post-GAS outcomes in their study, contrasting with the findings of most studies of transgender women.

Poorer QoL in transgender women compared to transgender men post-GAS has been suggested to be due to lower rates of acceptance of transgender women in society compared to transgender men, and this was highlighted both in France [26] and in Iran [34]. Studies have shown that transgender women are disproportionately affected by violence and discrimination. The National Center for Transgender Equality’s 2015 U.S. Transgender Survey [35] reported that 47% of transgender women experienced severe mistreatment in areas like employment, housing, and healthcare compared to 30% of transgender male respondents. Furthermore, physiological factors, such as skeletal or vocal characteristics that persist post-surgery, may contribute to increased visibility, potentially heightening the risk of discrimination and violence for transgender women relative to transgender men [36]. Other studies have commented on the hypersexualization of transgender women leading to fetishization and objectification [37,38]. A lower degree of social stigmatization in females adopting cross-gender behaviors has additionally been suggested as a driving factor in the recent increase in the ratio of transgender men to transgender women in gender clinics [39]. These external psychosocial stressors likely represent significant confounding variables that complicate the assessment of surgical efficacy across different gender populations.

### 4.4. Psychopathology as a Predictor

Agarwal et al. [8] does not mention the severity of psychopathology, only the presence of mental health conditions. Agarwal et al.’s assertion that those with existing mental health conditions have poorer body image scores pre-operatively but greater improvement post-operatively [8] lies in contrast to other studies [20,40] where higher psychopathology predicts worse post-operative functioning. It bears noting that Agarwal et al. [8] include only transgender men with chest reconstruction and not genital surgery.

### 4.5. Recommendations and Possible Predictors of Good Outcomes

As detailed in Table 2, the studies suggested that younger age, better education, improvement in secondary sexual characteristics and a supportive environment are predictors of good outcomes. Most studies however did not report on predictors of good outcomes, and among those which did report on these, there was little agreement on the predictors. Other studies did seem to suggest that non-homosexual orientation [24] and higher psychopathology pre-GAS were associated with worse outcomes. The importance of a supportive psychosocial environment has also been noted to be important [41].

The findings from this search underscore the need for enhanced pre-operative assessments for patients undergoing GAS, ensuring robust psychosocial support and perhaps closer monitoring of transgender women. The non-linear response of outcomes post-GAS and the ‘honeymoon period’ warns clinicians to avoid being overly optimistic about short-term improvements in their patients 1 year post-GAS and to continue longer term follow-up.

Future prospective studies are needed to better ascertain outcomes post-GAS and to better understand possible predictors for good outcomes. We advocate for outcome measures to be taken at both pre- and post-GAS and for follow-up times to be at least longer than the 1-year ‘honeymoon period’. Further qualitative studies into the differences in transgender women and transgender men post-GAS would be helpful to understand and explain outcome differences. There is unfortunately still a dearth of studies which measure objective psychiatric outcomes, such as admission or suicide rates post-GAS, with conflicting outcomes from the three studies included.

### 4.6. Strengths

The strengths of this review include a methodology which follows PRISMA guidelines. Our methodology emphasizes cohort studies and specifically excludes cross-sectional studies or studies which do not include a pre-GAS measurement. This helps establish stronger evidence of change over time, which is especially pertinent when outcomes such as QOL and psychological functioning often have many contributing factors. In addition, the inclusion of long-term cohort studies allows for the identification of trends in outcomes beyond a single time point.

### 4.7. Limitations

In our review, the exclusion of cross-sectional and cohort studies which did not take pre-operative measurements resulted in a smaller number of studies included. Comparison of outcomes between studies was challenging, as the sample of each study differed in the type of GAS and the ratio of transgender women or transgender men and may not have been directly comparable. Studies included often made limited use of objective psychiatric outcome measures, such as hospitalization rates or suicide attempts. The findings on psychological functioning were based mostly on self-reported QoL, which can be subjective and influenced by many external factors. Given the lack of available data, there may have been limited consideration of contextual factors such as differences in the degree and type of psychosocial support, differences in healthcare system and ease of access to transgender-affirming mental health care [42].

A limitation of this review is the conceptual grouping of diverse procedures under the broad term ‘GAS’; these include facial, chest and genital surgeries which may have distinct psychological, social, and physical impacts. Additionally, although cohort studies are utilized, the absence of matched control groups across several included studies precludes a definitive causal attribution of observed outcomes to surgical intervention alone. The studies span different cultural contexts and decades; the evolution of diagnostic criteria and differences in societal acceptance and healthcare accessibility make direct comparison across time periods a methodological constraint.

## 5. Conclusions

In conclusion, this review highlights the complex and varied mental health outcomes observed in transgender individuals following GAS. While initial improvements in QoL and reductions in gender dysphoria are reported, it remains uncertain whether these benefits are sustained over time. Findings on psychiatric outcomes come from a limited number of studies and further research is essential before recommendations can be made. Further research to explore differences in outcomes between transgender women and transgender men populations, with an emphasis on long-term follow-up, would better inform current clinical practice. Identifying the factors that influence these outcomes is crucial to provide tailored support and pre-operative mental health care, ultimately optimizing long-term success and improving mental health outcomes for the transgender population.

## Figures and Tables

**Table 2:** Possible predictors of outcomes of included studies.

No.	First Author, Year	Possible Predictors
1	Agarwal et al., 2018 [8]	Those with preexisting mental health conditions had, on average, poorer body image scores pre-operatively (2.9 compared to 2.4; *p* < 0.05). Having pre-existing mental health conditions predicted greater improvement from pre-operative to post-operative scores. No statistically significant correlation between post-op BREAST-Q scores and age, sexual orientation, gender identity, education level, employment status, or income.
2	Bränström et al., 2020 [17]	No mention.
3	da Silva et al., 2016 [13]	All results were controlled for variations in demographic characteristics, without significant results.
4	Dallas et al., 2021 [18]	Participants with a history of prior psychiatric emergencies or feminizing transition were at higher risk of suicide attempts post-GAS.
5	Simonsen et al., 2015 [9]	No mention.
6	De Vries et al., 2014 [15]	MTF had higher satisfaction over time with primary sex characteristics than transgender men, though none of the transgender men had undergone phalloplasty due to the waiting list or a desire for an improvement in surgical techniques. Greater improvements in secondary sex characteristics were correlated with higher subjective well-being at the final assessment. Participants with supportive families reported positive social experiences and had overall well-being comparable to their same-age peers.
7	Lindqvist et al., 2017 [12]	No mention.
8	Cohen-Kettenis, 1997 [19]	No mention.
9	Smith, Y. L. S. et al., 2001 [11]	No mention.
10	B. Udeze et al., 2008 [10]	No mention.
11	S. Naeimi et al., 2019 [14]	Younger age and better education were significantly associated with increase in mean score of total physical health on SF-36, whereas only better education was significantly associated with increase in mean score of total psychological health.
12	Smith, Y. L. S. et al., 2005 [20]	Non-homosexual orientation, higher psychopathology, and dissatisfaction with secondary sex characteristics at assessment were associated with poorer post-operative functioning. Transgender men showed a higher risk of dropping out and high psychopathology.
13	Chaovanalikit et al., 2022 [21]	No mention.
14	Inga Becker-Hebly et al., 2020 [16]	No mention.

**Table 3:** Newcastle–Ottawa Scale (cohort) for the included studies.

Study	Selection	Comparability	Outcome	NOS Score
Agarwal et al., 2018 [8]	***	*	-	4
Bränström et al., 2020 [17]	***	*	***	7
da Silva et al., 2016 [13]	***	*	**	6
Dallas et al., 2021 [18]	***	-	***	6
Simonsen et al., 2015 [9]	***	-	***	6
De Vries et al., 2014 [15]	***	*	**	6
Lindqvist et al., 2017 [12]	***	-	*	4
Cohen-Kettenis et al., 1997 [19]	***	-	***	6
Smith, Y. L. S. et al., 2001 [11]	***	-	***	6
B. Udeze et al., 2008 [10]	***	-	**	5
S. Naeimi et al., 2019 [14]	***	-	**	5
Smith, Y. L. S. et al., 2005 [20]	***	-	**	5
Chaovanalikit et al., 2022 [21]	***	-	*	4
Inga Becker-Hebly et al., 2020 [16]	***	-	*	4

Each ‘*’ refers to the number of stars per section of the Newcastle-Ottawa Scale (cohort), ‘-’ indicates no stars. Overall scores of 7–9 indicate high quality, 4–6 indicate fair quality, and 0–3 indicate poor quality.

## Data Availability

Original data used in this research belongs to the respective studies cited.

## Supplementary Materials

The following supporting information can be downloaded at: https://www.mdpi.com/article/10.3390/jcm15062213/s1, Table S1: PRISMA Checklist; Table S2. Complete Search Strings and Medical Subject Headings (MeSH) for All Databases.
